# Expression and characterization of *Pantoea* CO dehydrogenase to utilize CO-containing industrial waste gas for expanding the versatility of CO dehydrogenase

**DOI:** 10.1038/srep44323

**Published:** 2017-03-14

**Authors:** Eun Sil Choi, Kyoungseon Min, Geun-Joong Kim, Inchan Kwon, Yong Hwan Kim

**Affiliations:** 1 School of Materials Science and Engineering, Gwangju Institute of Science and Technology (GIST), Gwangju 61005, Republic of Korea; 2 Department of Biological Science, College of Natural Sciences, Chonnam National University, Gwangju 61186, Republic of Korea; 3 School of Energy and Chemical Engineering, UNIST, Ulsan 44919, Republic of Korea

## Abstract

Although aerobic CO dehydrogenases (CODHs) might be applicable in various fields, their practical applications have been hampered by low activity and no heterologous expression. We, for the first time, could functionally express recombinant PsCODH in *E. coli* and obtained a highly concentrated recombinant enzyme using an easy and convenient method. Its electron acceptor spectra, optimum conditions (pH 6.5 and 30 °C), and kinetic parameters (*k*_cat_ of 12.97 s^−1^, *K*_m_ of 0.065 mM, and specific activity of 0.86 Umg^−1^) were examined. Blast furnace gas (BFG) containing 20% CO, which is a waste gas from the steel-making process, was tested as a substrate for PsCODH. Even with BFG, the recombinant PsCODH retained 88.2% and 108.4% activity compared with those of pure CO and 20% CO, respectively. The results provide not only a promising strategy to utilize CO-containing industrial waste gases as cheap, abundant, and renewable resources but also significant information for further studies about cascade reactions producing value-added chemicals via CO_2_ as an intermediate produced by a CODH-based CO-utilization system, which would ultimately expand the versatility of CODH.

Carbon monoxide (CO), which is a pollutant in the atmosphere, is massively emitted through both natural (e.g., production by plants and volcanic activity) and artificial processes (e.g., incomplete combustion of fuels and industrial processes)^1^,^2^,^3^. Even though CO is normally fatal to living organisms, certain microorganisms are capable of utilizing CO as a carbon and energy source to produce useful organic compounds as metabolites^4^ and thus such microorganisms have been widely used to construct a CO-utilization system for producing fuels and chemicals^5^,^6^. CO dehydrogenase (CODH, E.C. 1.22.99.2), which is a key enzyme in CO metabolism^1^,^5^,^6^,^7^,^8^, usually catalyzes the oxidation of CO to CO_2_ and thus might be applicable for transforming hazardous CO into non-toxic CO_2_ in various fields. However, applications for these enzymes have not been intensively explored. Although structural information and the catalytic mechanism of anaerobic CODHs have been intensively researched^9^,^10^, practical applications have been hampered by the lack of oxygen tolerance. For aerobic CODHs retaining their catalytic activity in the presence of oxygen, broad applications have been limited due to their relatively low activity and no heterologous expression for convenient production.

A considerable amount of CO-containing waste gases are generated from several industrial processes. Table S1 summarizes various CO-containing waste gases from a steel-making process at POSCO with different compositions. Currently, these waste gases are combusted as fuels on site, but these gases have not only a risk of explosion but also a poor fuel-efficiency owing to the contained inert gas such as N_2_. Accordingly, CO-containing industrial waste gases have received much attention, because they might be cheap, abundant and renewable resources for producing value-added chemicals and therefore microbial CO-utilization using industrial waste gases as the feedstock has been attempted^5^,^6^. In this study, we aimed to extend the scope of microbial CO-utilization towards enzymatic CO-utilization for higher specificity and productivity. Given that (i) an aerobic CODH is able to catalyze CO in the industrial waste gases to CO_2_ and (ii) CO_2_ is often used as a building block for producing useful chemicals (e.g., urea, methanol, formate, and acetone) through chemical and biological routes with environmental and economic benefits^11^, constructing a CODH-based CO-utilization system via CO_2_ as an intermediate was our challenge. Moreover, aerobic CODHs could be practically used for treating hazardous CO in daily life. For example, aerobic CODHs immobilized on the filter of a cigarette seem to effectively convert hazardous CO generated during smoking into non-toxic CO_2_.

Herein, we, for the first time, reported the heterologous expression of an aerobic CODH in *E. coli* for producing a highly concentrated recombinant enzyme using an easy and convenient process for practical applications. In particular, anaerobic CODH obtained from *Pantoea* species YR343 (PsCODH) was newly discovered through genome-mining and phylogenetic analysis. To characterize the recombinant PsCODH, electron acceptor spectra, optimum conditions, and kinetic parameters were determined. Furthermore, the feasibility of PsCODH was evaluated for constructing the CO-utilization system with a CO-containing waste gas from the steel-making process as cheap, abundant, and sustainable feedstock.

## Results

### A newly discovered aerobic CODH from the Pantoea species YR343 (PsCODH) was overexpressed in *E. coli*

Although aerobic CODHs are significant for developing enzymatic CO-utilization systems, the heterologous expression of an aerobic CODH has not been reported up to date. In order to discover aerobic CODHs that could be expressed in *E. coli*, we explored proteins from diverse microorganisms listed in the UniProt Knowledgebase (UniProtKB, http://www.uniprot.org/uniprot/), which is the most widely used database for functional information on proteins and includes accurate and detailed annotations. To search for potential CODHs from UniProtKB, we used the aerobic CODH from *Oligotropha carboxidovorans* (OcCODH) as a reference, since OcCODH has been one of the most intensively researched aerobic CODHs and has structural information is available (PDB ID: 1N5W). OcCODH is a dimer of heterotrimers consisting of CoxS with two Fe_2_S_2_ clusters, CoxM with non-covalently bound FAD, and CoxL containing a molybdopterin active site. Therefore, several keywords including aerobic CODH, aerobic *coxS*, aerobic *coxM*, and aerobic *coxL* were used, and several hundreds of hits were resulted (data not shown).

**Figure 1:**
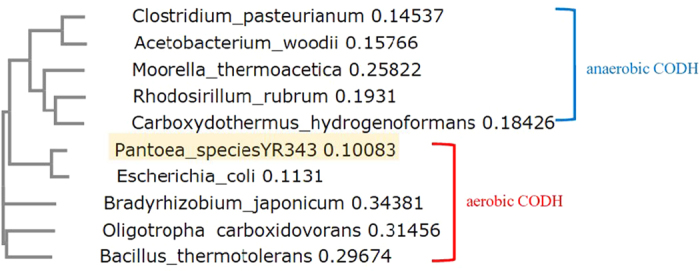
Phylogenetic tree comparing the CODH from the *P.* species YR343 with previously reported anaerobic and aerobic CODHs.

Then, we assumed that microorganisms phylogenetically close to *E. coli* would result in good functional expression of their genes in *E. coli*. In particular, we focused on microorganisms between *E. coli* and *Bacillus* species in the phylogenetic trees. The *Bacillus* species are obligate aerobes or facultative anaerobes, and their proteins also usually express well in *E. coli*^12^,^13^,^14^,^15^. Thus, CODHs from species between *E. coli* and *Bacillus* were investigated from the CODHs found in our UniProtKB search. As a result, a CODH from the *Pantoea* species YR343 (PsCODH) was chosen because it was (i) closer to the CODH from *E. coli*, a commonly used host strain for overexpression, in the phylogenetic tree (Fig. 1); (ii) it was classified as an Enterobacteriaceae similar to *E. coli*; and (iii) it was phylogenetically located on the border between aerobic and anaerobic CODHs (Fig. 1), thereby expecting that PsCODH may partially share features of anaerobic CODH that are heterologously expressed in *E. coli*^16^,^17^.

**Figure 2:**
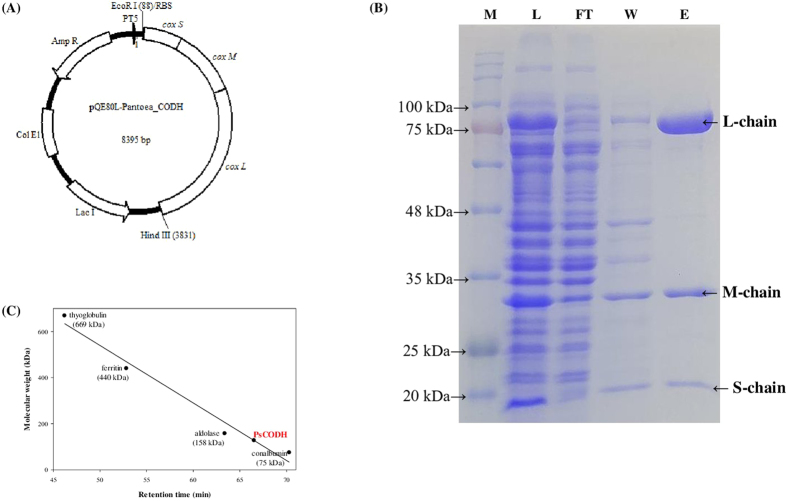
(**A**) Schematic illustration of the expression vector (Amp R: ampicillin-resistance gene, large, medium, and small chain of the CODH gene sequence encoding *cox L, cox M*, and *cox S*, Lac I: Lac I repressor gene sequence), **(B)** SDS-PAGE (M: pre-stained protein standard, L: cell lysate, FT: flow through fraction, W: wash fraction with 20 mM imidazole, E: eluted protein with 250 mM imidazole) of the purified recombinant PsCODH using a 12 % acrylamide gel under denaturing conditions, and **(C)** determination of the molecular mass of the gross purified recombinant PsCODH using size exclusion chromatography. Thyroglobulin (699 kDa), ferritin (440 kDa), aldolase (158 kDa), and conalbumin (75 kDa) were used as reference proteins.

For the overexpression of PsCODH in *E. coli*, genes encoding the L, M, and S chains of PsCODH were cloned into the pQE80L vector. A hexahistidine-tag was added to the C-terminus of the L chain for purification as shown in Fig. 2(A). After overexpression and purification using Ni-NTA resin, the homogeneity of the recombinant PsCODH was investigated by SDS-PAGE as shown in Fig. 2(B). The molecular weights of the L, M, and S chains of the recombinant PsCODH were estimated to be 78.0, 33.7, and 19.6 kDa, respectively. To calculate the molecular mass of the gross recombinant PsCODH, size exclusion chromatography (SEC) was performed. As shown in Fig. 2(C) and Fig. S1, the molecular weight of the gross recombinant PsCODH was estimated to be 127.4 kDa. Additionally, heterologous expression enabled us to obtain highly concentrated recombinant CODH with a yield of up to 4.8 mg from a 300 mL culture.

Furthermore, *in vivo* and *in vitro* FAD reconstitution were performed to obtain functional recombinant PsCODH, and the reconstitution was evaluated by determining the ratio of A_450_/A_280_^18^. As a result, the A_450_/A_280_ of the recombinant PsCODH without reconstitution was 0.71 ± 0.11, whereas *in vivo* reconstitution increased the A_450_/A_280_ to 1.81 ± 0.50. For *in vitro* reconstitution, the A_450_/A_280_ was about 0.63 ± 0.23. Consequently, *in vivo* reconstitution was the most effective for FAD-reconstitution and thus additional experiments were performed with the *in vivo* reconstituted recombinant PsCODH in this study.

### Biochemical characterization and kinetic analysis

**Figure 3:**
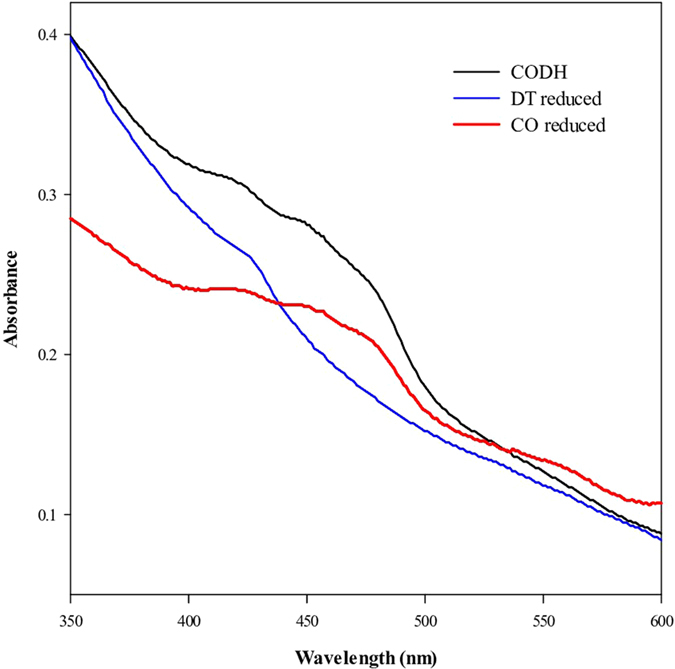
UV-vis absorption spectra of the recombinant PsCODH. Spectra are of the native PsCODH (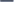), the CO-reduced PsCODH (2 min CO purging [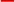]), and the sodium dithionite (DT)-reduced PsCODH (1 min after mixing [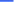]). The spectra were recorded in elution buffer containing 250 mM imidazole (pH 8.0). The base-line was adjusted using an elution buffer before recording.

For validating the functionality of the recombinant PsCODH, the extent of CO-driven and DT-driven PsCODH reduction were compared^19^. As shown in Fig. 3, CO-purging for 2 min reduced the absorbance at 450 nm (ε_FAD, 450 nm_ = 11.3 mM^−1^ cm^−1^) by approximately 71.8 % compared to the DT-reduced PsCODH, which demonstrated that 71.8 % of the recombinant PsCODH was functional and was able to catalyze CO to CO_2_. Further incubation of the recombinant PsCODH with CO for 30 min resulted in complete reduction, which was similar to the reduction of the DT-reduced form. This result indicated that a certain amount of non-functional PsCODH was slowly reduced, probably due to intermolecular electron transfer from the reduced functional PsCODH^19^.

We already evaluated the functionality of the recombinant PsCODH by monitoring the reduced FAD (Fig. 3), but the CO-oxidizing activity of CODH was typically assayed with artificial electron acceptors and often depended on the type of electron acceptor used^20^. Accordingly, the electron acceptor spectrum of the recombinant PsCODH was examined. As shown in Table 1, the recombinant PsCODH used methylene blue (MB), phenazine methosulfate –nitroblue tetrazolium chloride (PMS-NBT), thionine acetate (TA), benzyl viologen (BV), and methyl viologen (MV) as electron acceptors to varying extents. Even if MV was the most widely used electron acceptor for assaying anaerobic CODH, it was less effective for aerobic recombinant PsCODH and exhibited only 22.0 % and 8.6 % activity relative to MB and PMS-NBT, respectively. PMS-NBT and TA have not been used for assaying anaerobic CODH, but these compounds were effective for aerobic recombinant PsCODH, probably due to the different redox center in the active site. The aerobic CODH usually contains a [Mo- Fe-S] cluster, whereas anaerobic CODH contains an [Ni-Fe-S] cluster in its activecenter^17^,^21^. Among the tested electron acceptors, PMS-NBT was the most effective for accepting electrons from recombinant PsCODH.

**Figure 4:**
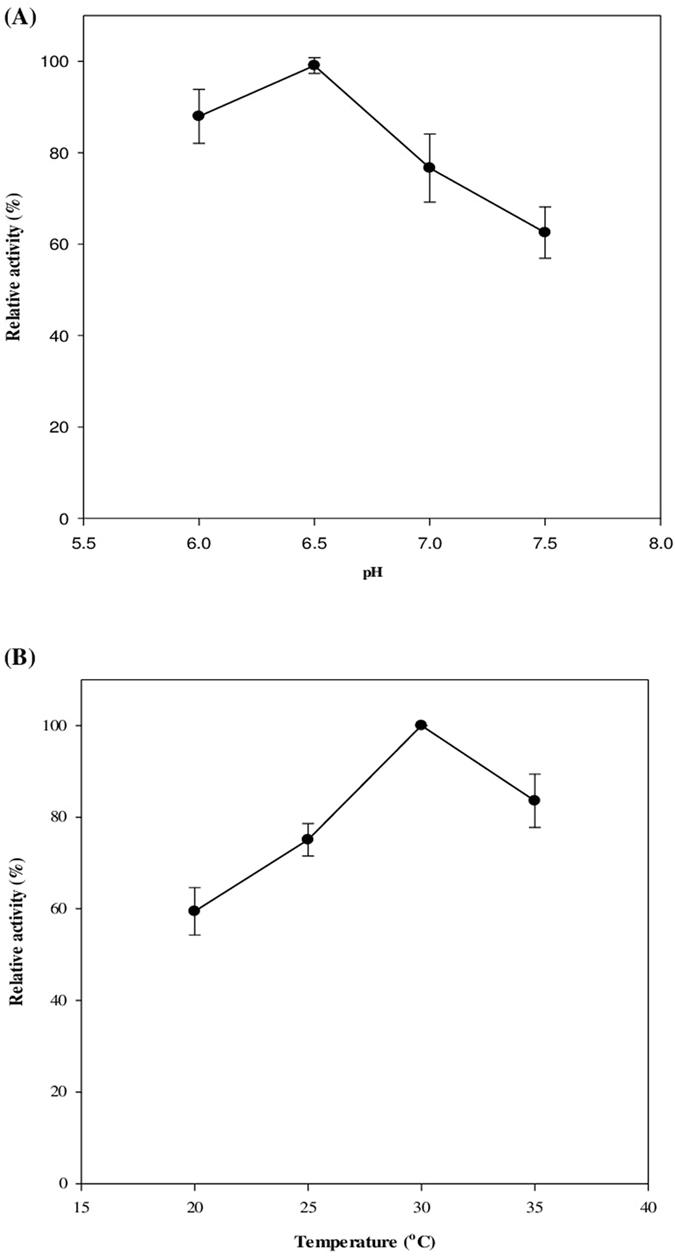
Optimum **(A)** pH and **(B)** temperature of the recombinant PsCODH using 20 μM thionine acetate (TA) as the electron acceptor. The optimum pH was examined at 30 °C using 200 mM phosphate buffer (pH 6.0–pH 7.5). The optimum temperature was determined at the optimum pH shown in Fig. 4A. The experiments were performed in triplicate.

Before determining the kinetic parameters, the optimum pH and temperature for CO-oxidizing activity were investigated. Figure 4 describes that the optimum pH and temperature were 6.5 (200 mM, potassium phosphate buffer) and 30 °C, respectively. The optimum parameters for CO-oxidation were not far from the growth conditions for the *Pantoea* species, i.e., typically 25–30 °C and pH 7^22^,^23^.

**Figure 5:**
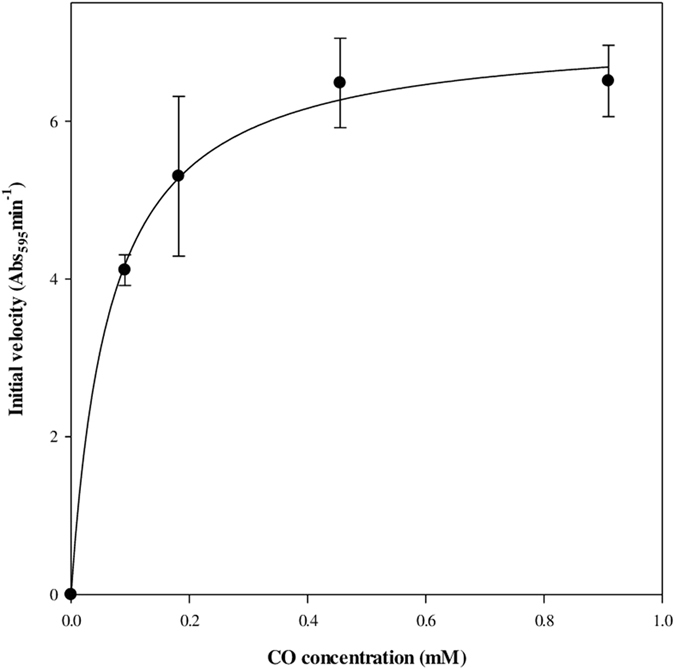
Kinetic assay of the recombinant PsCODH for CO oxidation. The reaction was performed at pH 6.5 (200 mM, potassium phosphate buffer) and 30 °C with thionine acetate (TA, 20 μM) as the electron acceptor. The initial velocity was monitored by reduction of the electron acceptor at 595 nm using a UV-visible spectrophotometer. The solid line is the fit with the Michaelis-Menten equation (R^2^ = 0.9972). The experiments were performed in triplicate.

As shown in Fig. 5, the initial TA reduction velocity depended on the CO concentration under optimum conditions. The initial velocity exhibited a rectangular-hyperbolic increase, and the data was fitted with a Michaelis-Menten equation with a high regression coefficient (R^2^ = 0.9972), thereby indicating a *k*_cat_ of 9.31 s^−1^ and a K_m_ of 0.065 mM.

### PsCODH utilized CO-containing waste gas from the steelmaking process

**Figure 6:**
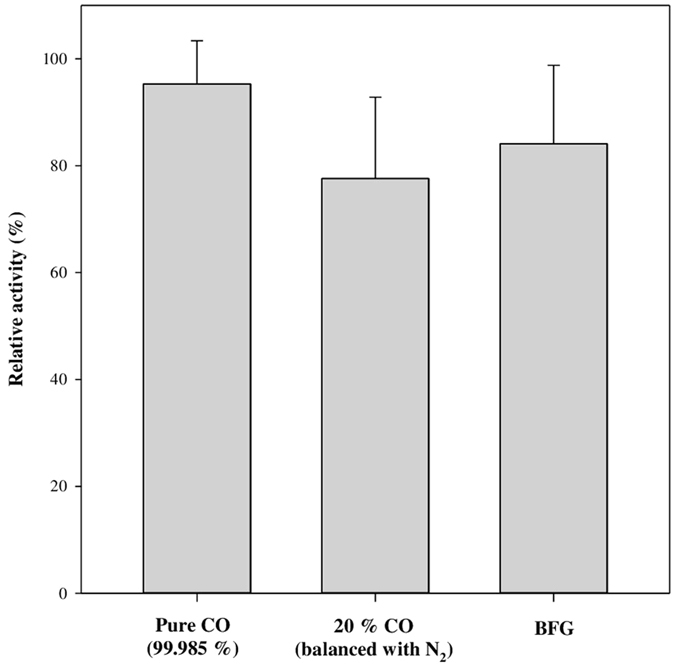
Relative activity of the recombinant PsCODH using BFG, a kind of CO-containing waste gas from the steelmaking process. The activity was assayed at pH 6.5 (200 mM phosphate buffer) and 30 °C using thionine acetate as the electron acceptor (20 μM). The relative activity was calculated based on the percentage of activity when using pure CO as the substrate. The experiments were performed in triplicate

In order to evaluate the feasibility of recombinant PsCODH for constructing an enzymatic CO-conversion system with industrial waste gas as the feedstock, the specific activity was compared with pure CO, 20 % CO balanced with N_2_, and BFG containing 22 % CO and 20 % CO_2_ as the substrate. Although we aimed to compare all waste gases in Table S1, COG and LDG were associated with a risk of explosion and thus were not suitable for testing in the laboratory despite the fact that LDG contained the highest amount of CO, up to 65 %, which was a promising feedstock for enzymatic CO utilization. Thus, only BFG was evaluated in this study. As a result, BFG retained 88.2 ± 7.4 % activity relative to that of pure CO (Fig. 6). In addition, BFG maintained 108.4 ± 7.6 % of relative activity compared to that of 20 % CO. The results implied that (i) H_2_ and N_2_ in BFG did not negatively influence CODH activity and (ii) the recombinant PsCODH was not inhibited by CO_2_ as a product from PsCODH catalysis. Consequently, the recombinant PsCODH was found to be a promising candidate for developing an enzymatic CO-conversion system using industrial waste gases as a cheap and sustainable feedstock.

## Discussion

Although aerobic CODHs are essential for developing enzymatic CO-utilization systems for producing useful chemicals and fuels from CO, the heterologous expression of an aerobic CODH has not been reported up to date. To the best of our knowledge, this is the first report about the heterologous functional expression of an aerobic CODH in *E. coli*. Through genome-mining and phylogenetic analysis, we newly identified an aerobic CODH from the *Pantoea* species YR343 (PsCODH) and characterized the electron acceptor spectra, optimum pH and temperature, kinetic parameters, and specific activity of the PsCODH. SDS-PAGE and SEC in Fig. 2 represented that PsCODH may exist as a heterotrimer (LMS) that is distinct from OcCODH, which exists as an (LMS)_2_ hexamer^24^,^25^ and is used as the reference CODH for genome-mining in this study. In the near future, we are planning to study the 3-dimensional structure of PsCODH to provide structural information regarding aerobic CODHs that can be expressed in *E. coli* and to provide insight into the catalytic mechanisms of the electron transfer pathway. As shown in Table 1, PsCODH exhibited broad electron acceptor spectra to varying extents. Comparing CODHs from *Carboxydothermus hydrogenoformans*^17^ and *O.carboxydothermus*^21^, the metal cluster in the redox center might affect the preference of the electron acceptor and thus the structural validation of PsCODH could explain not only the electron acceptor preference but also the role of the metal cluster in the catalytic mechanism. The kinetic parameters of PsCODH were compared to the parameters of aerobic CODHs that were previously reported (Table 2). CODHs from *Bradyrhizobium japonicum*^24^ and *O.carboxydothermus*^19^, which are capable of chemolithoautotrophic growth using CO as a sole carbon and energy source, oxidized CO to CO_2_ to varying extents with various electron acceptors^19^,^24^. Even though it is unclear whether *P.* species YR 343 utilizes CO as the sole carbon and energy source, the recombinant PsCODH exhibited a specific activity of 0.86 Umg^−1^. The purified anaerobic CO-oxidizing:H_2_-evolving enzyme complex from *C. hydrogenoformans* was slightly inhibited by the substrate CO^9^, whereas aerobic recombinant PsCODH did not exhibit substrate inhibition at all. Given that the recombinant PsCODH used in this assay was approximately 71.8 % active (Fig. 3), the corrected *k*_cat_ value for the fully functional PsCODH seemed to be maximized up to 12.97 s^−1^ with TA as the electron acceptor^19^.

Various industrial processes discharge considerable amounts of CO into the atmosphere. For example, POSCO, which is the most competitive steel-maker in the world, emits huge amounts of CO-containing waste gases with different compositions (Table S1). Currently, most waste gases are combusted as fuels at POSCO, but the inert gases in the waste gas are impediments to fuel efficiency. Accordingly, it would be helpful to establish methods for using waste gases for other purposes. Therefore, we focused on evaluating whether waste gases may be cheap and sustainable resources for CO-utilization for producing value-added chemicals. Figure 6 implies that (i) H_2_ and N_2_ in BFG did not negatively influence CODH activity and (ii) the recombinant PsCODH was not inhibited by CO_2_ that was a product from PsCODH catalysis. Consequently, the recombinant PsCODH was found to be a promising candidate for developing an enzymatic CO-conversion system using industrial waste gases as cheap, abundant, and sustainable feedstock for producing value-added chemicals. Since CODH is the key enzyme for CO-metabolism in CO-utilizing microorganisms, research on CODH has been focused on the catalytic mechanisms and structural information. However, practical applications of CODH to produce value-added chemicals have not been thoroughly explored to date. The results described in this study might contribute to expanding the versatility of CODH towards practical uses even under aerobic conditions. For example, aerobic CODH immobilized on a cigarette filter could convert toxic CO generated from smoking to non-toxic CO_2_. Furthermore, the results might open a new door to cascade reactions consisting of CO conversion to CO_2_ and CO_2_ conversion to useful fuels and chemicals via CO_2_ as an intermediate. Accordingly, we plan on performing cascade reactions that produce formate, which is the most beneficial bulk chemical obtained from CO_2_ from an environmental and economic perspective^11^, by utilizing BFG as a feedstock in the near future.

## Methods

### Chemicals

MV, BV, MB, NBT, PMS, TA, FAD, sodium molybdate, and sodium dithionite were purchased from Sigma-Aldrich (St. Louis, MO, USA). BugBuster^®^ and Ni-NTA resin were bought from Novagen (Billerica, MA, USA) and Qiagen (Valencia, CA, USA), respectively. *Pfu* polymerase, *EcoR*I, *Hind*III, and *Dpn*I were purchased from Enzymonics (Daejeon, Korea). As a CO-containing waste gas from the steelmaking process, BFG was prepared from Seongkwang (Seoul, Korea). Unless otherwise stated, all chemicals were used at the highest grade available without further purification.

### Cloning and overexpression of PsCODH in *E. coli*

Genes encoding the large (*coxL*; GenBank accession: EJM96790), medium (*coxM*; GenBank accession: EJM96789), and small (*coxS*; GenBank accession: EJM96790) chains of PsCODH were synthesized by GenScript (Piscataway, NJ, USA). The gene sequences were codon-optimized for expression in *E. coli* and were then cloned into the pQE80 vector between the *EcoR*I and *Hind*III sites, which generated pQE80-Ps_L, pQE80-Ps_M, and pQE80-Ps_S, including *coxL, coxM*, and *coxS*, respectively. For the construction of pQE80-Ps_SML containing the large, medium, and small chains of PsCODH, we performed restriction-free (RF) cloning^26^,^27^. To amplify the medium and small chains, a polymerase chain reaction (PCR) was performed using pQE80-Ps_M and pQE80-Ps_S as templates with the primers listed in Table S2, respectively. To construct pQE80-Ps_ML containing the large and medium chains of PsCODH, RF cloning was performed with the PCR products from pQE80-Ps_M and pQE80-Ps_L as a primer and a template, respectively. To construct pQE80-Ps_SML containing the large, medium, and small chains of PsCODH, RF cloning was performed with the PCR products from pQE80-Ps_S and pQE80-Ps_ML as a primer and a template, respectively. The RF cloning product was purified, incubated with *Dpn*I at 37 °C for 2 hours, and then transformed into competent *E. coli* Top10 (DE3) cells generating Top10_pQE80-Ps_SML-expressing cells. The mixture of transformed cells was spread on a Luria-Bertani (LB) agar plate containing 100 μg mL^−1^ ampicillin. The gene sequences of PsCODH obtained from several colonies were verified by Macrogen (Seoul, Korea).

For overexpression, Top10_pQE80-Ps_SML cells were grown in LB medium (300 mL in 1 L flask) containing 50 μg mL^−1^ ampicillin and 1 mM sodium molybdate at 37 °C and 200 rpm. When the optical density of cells at 600 nm reached approximately 1.0, 1.0 mM isopropyl-β-d-thiogalactopyranoside (IPTG) and 100 μM FAD were added for induction and *in vivo* reconstitution, respectively. Then, the temperature was lowered to 25 °C. After a 24-hour cultivation, the cells were harvested by centrifugation (12,000 rpm, 30 min, 4 °C), and the cell pellet was stored at −70 °C before use^18^,^28^.

### *In vitro* reconstitution and purification of recombinant PsCODH

For the cell lysis, the cell pellet was suspended in BugBuster^®^ (1 g cell pellet in 5 mL BugBuster^®^). After 1 hour of incubation on ice with shaking at 20 rpm, the cell debris were removed by centrifugation (12,000 rpm, 30 min, 4 °C). Then, 100 μM FAD was added to the crude cell lysate for *in vitro* reconstitution, and the mixture was incubated on ice for 1 hour with shaking at 20 rpm. Then, the lysate was loaded onto Ni-NTA resin, the column was washed with buffer containing 20 mM of imidazole, and finally 3 mL of the purified recombinant PsCODH was obtained. The purified recombinant PsCODH was quantified by Bradford assay using bovine serum albumin as the standard^18^.

The molecular masses of the L, M, and S chains of PsCODH were identified by sodium dodecyl sulfate polyacrylamide gel electrophoresis (SDS-PAGE) using a 12 % acrylamide gel under denaturing conditions. All protein bands were stained with Coomassie Blue (Bio-Rad, Hercules, CA, USA) for visualization^18^. The molecular mass of the gross recombinant PsCODH was estimated by SEC using a Superdex 200 Increase 10/300 GL column (GE Healthcare Life Sciences, United Kingdom). The purified recombinant CODH was loaded onto the column and eluted at a flow rate of 0.2 mL min^−1^ with phosphate buffer (50 mM, pH 7.2) containing 150 mM NaCl. Thyroglobulin (669 kDa), ferritin (440 kDa), aldolase (158 kDa), and conalbumin (75 kDa) were used as reference proteins. The molecular mass of the gross recombinant PsCODH was calculated by comparing the migration time with those of the reference proteins^29^.

### Kinetic analysis

To evaluate electron acceptor spectra, MB (15.6 μM, ε_670nm_ = 30.5 mM^−1^ cm^−1^), PMS (100 μM) –NBT (50 μM, ε_540nm_ = 7.2 mM^−1^ cm^−1^), BV (1.0 mM, ε_600nm_ = 7.4 mM^−1^ cm^−1^), MV (10 mM, ε_578nm_ = 9.7 mM^−1^ cm^−1^), and TA (20 μM, ε_595nm_ = 42 mM^−1^ cm^−1^) were tested at pH 7.0 (200 mM phosphate buffer) and at 30 °C^10^,^19^,^24^,^30^,^31^. CODH activity was monitored by the CO-dependent reduction of each electron mediator as follows: Pure CO (99.985%) was purged into the headspace of a rubber septum-stopped cuvette containing each electron mediator and the recombinant PsCODH in phosphate buffer (200 mM, pH 7.0) for 2 min. After 30 min of incubation at 30 °C, the absorbance was measured using a UV-visible spectrophotometer (UV-1650PC, Shimadzu). The relative activity of each mediator was calculated based on the percentage of activity compared to the activity using MB as the electron acceptor^32^.

The optimum pH and temperature were determined using 20 μM TA as the electron acceptor. For the optimum pH, 200 mM phosphate buffer (pH 6.0–pH 7.5) was used at 30 °C. Then, the optimum temperature was investigated at the optimum pH shown in Fig. 3(A).

For calculating kinetic parameters, 0, 10, 20, 50, and 99.985 % of CO balanced using N_2_ were used. By Henry’s law: 0, 10, 20, 50, and 99.985 % CO in the headspace corresponded to 0, 0.091, 0.182, 0.455, and 0.909 mM CO in the aqueous phase at 30 °C, respectively. To measure the initial velocity, each concentration of CO was purged into the headspace of a rubber septum-stopped cuvette containing 20 μM TA in phosphate buffer (200 mM, pH 6.5) for 2 min. For saturating CO into the solution, the cuvette was incubated at 30 °C for 1 hour. Then, the recombinant PsCODH was injected using a syringe to initiate the reaction. The absorbance change at 595 nm was monitored at 30 °C using a UV-visible spectrophotometer. Kinetic parameters (*k*_cat_ and *K*_m_) were calculated from the Michaelis-Menten equation. To calculate the relative activity with CO-containing waste gas from the steelmaking process, the initial velocity with BFG was measured using the same procedure as described above.

## Additional Information

**How to cite this article**: Choi, E. S. *et al*. Expression and characterization of *Pantoea* CO dehydrogenase to utilize CO-containing industrial waste gas for expanding the versatility of CO dehydrogenase. *Sci. Rep.* **7**, 44323; doi: 10.1038/srep44323 (2017).

**Publisher's note:** Springer Nature remains neutral with regard to jurisdictional claims in published maps and institutional affiliations.

## Supplementary Material

Supplementary Information

## Figures and Tables

**Table 1:** Electron acceptor spectra of the recombinant PsCODH.

Electron acceptor	Relative activity (%)
Methyl viologen (MV)	22.0 ± 9.1
Benzyl viologen (BV)	8.8 ± 3.3
Methylene blue (MB)	100 ± 10.6
Phenazine methosulfate - nitroblue tetrazolium chloride (PMS-NBT)	256.7 ± 22.4
Thionin acetate (TA)	37.3 ± 3.5

The experiments were performed in triplicate.

**Table 2:** Kinetic parameters and specific activities of aerobic CODHs.

	*k*_cat_ (s^−1^)	K_m_ (mM)	Specific activity (U mg^−1^)*	Electron acceptor	Assay condition	References
*Bradyrhizobium japonicum*			0.058	PMS-NBT	pH 7.5	24
*Oligotropha carboxidovorans*	93.3	0.011		MB	pH 7.2, 30 °C	19
*Pantoea* species YR343	12.97	0.065	0.86	TA	pH 6.5, 30 °C	This study

^*^1 U of enzyme oxidized 1 μmol of CO to CO_2_ per minute.

The experiments were performed in triplicate.
