# Supplementary material for: Expression and characterization of Pantoea CO dehydrogenase to utilize CO-containing industrial waste gas for expanding the versatility of CO dehydrogenase
Source: Sci Rep. 2017 Mar 14;7:44323. doi: 10.1038/srep44323 (PMC5349547; doi:10.1038/srep44323)
Supplement: Supplementary Information [file srep44323-s1.doc]

Supplementary Information

**Expression and characterization of Pantoea CO dehydrogenase to utilize CO-containing industrial waste gas for expanding the versatility of CO dehydrogenase**

Eun Sil Choi^1,2a^, Kyoungseon Min^3a^, Geun-Joong Kim^2^, Inchan Kwon^1*^, and Yong Hwan Kim^3*^

^1^School of Materials Science and Engineering, Gwangju Institute of Science and Technology (GIST), Gwangju 61005, ^2^Department of Biology, Chonnam National University, Gwangju 61186, ^3^School of Energy and Chemical Engineering, UNIST, Ulsan 44919, Republic of Korea

^a^These authors contributed equally to this work.

**Table S1**. Composition of waste gas from steelmaking process at POSCO

|  | | **Coke Oven Gas**  **(COG)** | **Blast Furnace Gas**  **(BFG)** | **Lintz Donawitz Converter Gas**  **(LDG)** | **FINEX Off Gas**  **(FOG)** |
| --- | --- | --- | --- | --- | --- |
| **Component**  **(%)** | **CO** | 7 - 8 | 20 - 22 | 60 - 65 | 25 |
|  | **H_2_** | 56 - 58 | 3 - 4 | 0 - 2 | 14 |
|  | **N_2_** | 1.5 | 54 - 56 | 20 - 22 | 18 |
|  | **CO_2_** | 2.4 - 3 | 20 - 22 | 16 - 18 | 42 |
|  | **CH_4_** | 25 - 26 | - | - | 1 |
|  | **C_2_H_4_** | 2.2 – 5.2 | - | - |  |
|  | **O_2_** | 0.2 – 0.5 | - | 0.1 | 0.5 |
| **Production**  **(Nm^3^/h)** | | 235,000 | 1,860,000 | 130,000 | 390,000 |

**Table S2.** Primers used in this study

| **Purpose** | **Name** | **Sequence** |
| --- | --- | --- |
| *coxM* amplication | F1 | 5’-CGGATAACAATTTCACACAGAATTCATTAAAGAGGAGAAATTAACTATGAAGAGCTTCACCTACCAACGTG-3’ |
|  | R1 | 5’- CCCGCCGGTTTCTCGAACTTCATGTGTTAACCACGCGCTTCCGC– 3’ |
| *coxS* amplication | F2 | 5’ – CGGATAACAATTTCACACAGAATTCATTAAAGAGGAGAAATTAACTATGAGCAGCCAACACTTAACAACAC – 3’ |
|  | R2 | 5’ – GGGTTTTAACACGTTGGTAGGTGAAGCTCTTCATGATTCCAGGCCCTCCG – 3’ |


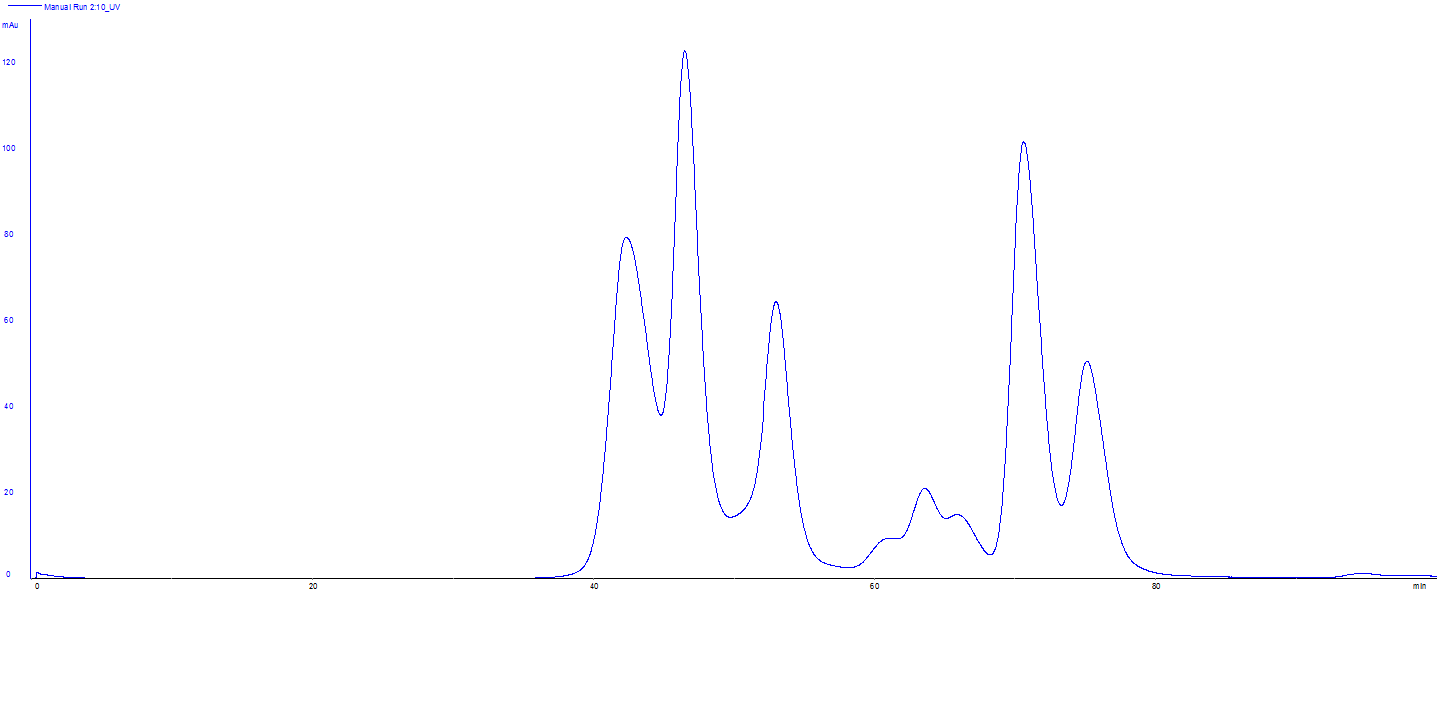


Thyroglobulin

(669 kDa)

Ferritin

(440 kDa)

Aldolase

(158 kDa)

Conalbumin

(75 kDa)

20

40

60

0

80


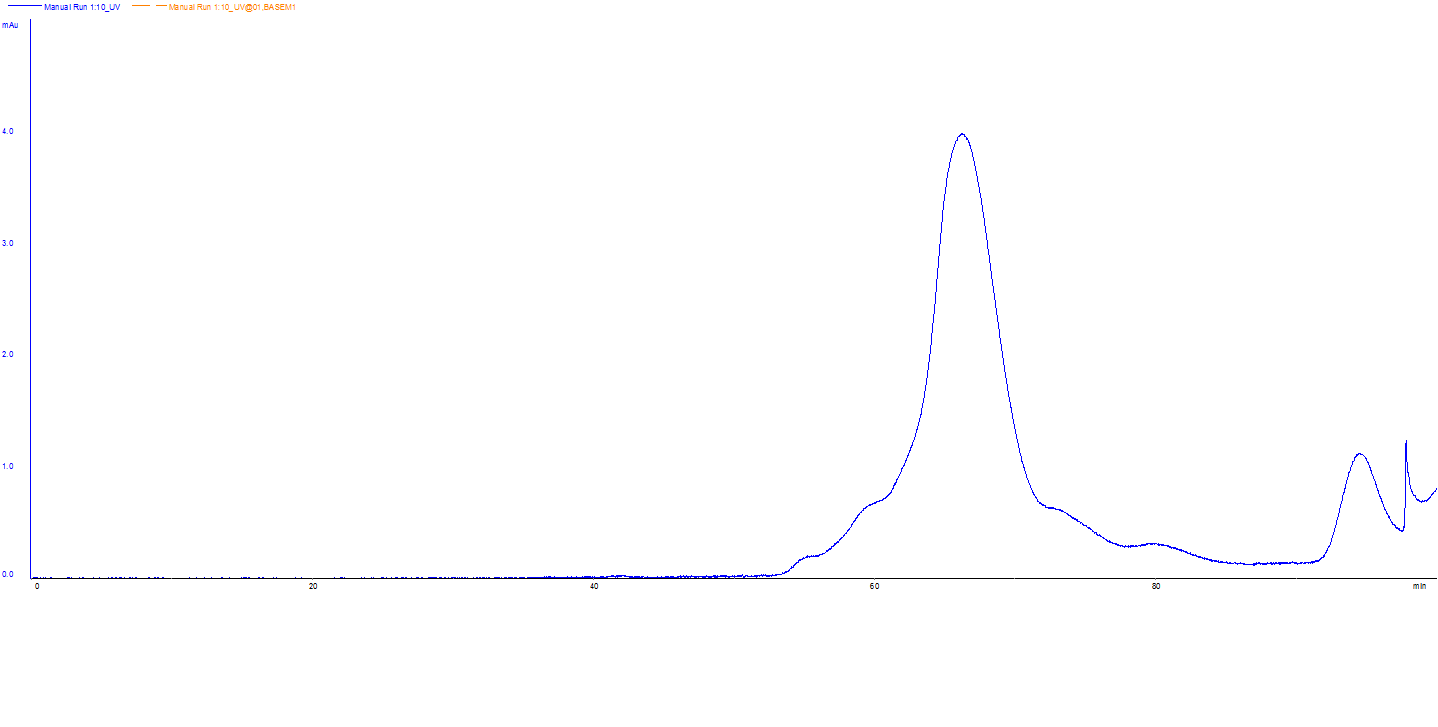


20

40

60

0

80

0

1

2

3

4

5

**Intensity (a.u.)**

**Intensity (a.u.)**

**Retention time (min)**

0

20

40

60

80

100

120

**PsCODH**

**(A)**

**(B)**

**Figure S1.** Size exclusion chromatography (SEC) of **(A)** reference proteins (thyroglobulin [669 kDa], ferritin [440 kDa], aldolase [158 kDa], and conalbumin [75 kDa]) and **(B)** PsCODH using a Superdex 200 Increase 10/300 GL column.
